# Clinical and epidemiological profile of tuberculosis in an urban area with high human development index in southeastern Brazil. Time series study

**DOI:** 10.1590/1516-3180.2016.0260210317

**Published:** 2017-08-21

**Authors:** Rodolpho Telarolli, Leonor Castro Monteiro Loffredo, Rosangela Maria Gasparetto

**Affiliations:** I Medical Doctor, Doctor and Adjunct Professor, Department of Biological Sciences, Universidade Estadual Paulista (UNESP), Araraquara (SP), Brazil.; II Medical Doctor, Doctor and Adjunct Professor, Department of Social Dentistry, Universidade Estadual Paulista (UNESP), Araraquara (SP), Brazil.; III Bachelor of Science and Nurse, Araraquara Special Health Service (SESA), Universidade de São Paulo (USP), Araraquara (SP), Brazil.

**Keywords:** Tuberculosis, Human development, Epidemiology, Risk factors, Acquired immunodeficiency syndrome

## Abstract

**CONTEXT AND OBJECTIVE:**

In the twenty-first century, tuberculosis remains a serious public health problem in Brazil. The aim here was to characterize tuberculosis in a municipality with a high human development index (HDI), based on clinical and epidemiological variables.

**DESIGN AND SETTING:**

Epidemiological study with analysis of incidence that included 533 new cases of tuberculosis in the municipality of Araraquara, São Paulo, reported to the Brazilian Notifiable Diseases Information System (SINAN) between 2002 and 2011.

**METHODS:**

To identify trends, this period was divided into two five-year periods (2002-2006 and 2007-2011). The incidence rates were compared using ratios and confidence intervals.

**RESULTS:**

The incidence of tuberculosis was 26.82 cases per 100,000 inhabitants, and decreased by 22% between the two periods, which was statistically significant. Cases were more prevalent among men (72.61%) and among adults between 30 and 59 years of age with non-specialized professions and low education levels. There was a statistically significant reduction in cases among individuals over 50. The age group with highest incidence was 50-59 years in the first period and 30-39 years in the second. Total recovery occurred in more than 70%. There was a reduction in the number of diagnoses made within primary care and an increase within public hospital care between the two periods. The most common coinfections were AIDS and hepatitis C.

**CONCLUSIONS:**

The incidence of tuberculosis in this municipality was lower than the national incidence, with a declining trend and a high cure rate, and the main coinfections were AIDS and hepatitis C.

## INTRODUCTION

Tuberculosis (TB) was declared a global emergency by the World Health Organization (WHO) in 1993. WHO has recommended directly observed treatment (short-course treatment), or DOTS, which focuses on detection, increasing the cure rate and decreasing patient withdrawal from treatment.^1^ DOTS was quickly adopted by the Brazilian government, and resulted in improved care for TB carriers. Between 1995 and 2012, 56 million people were successfully treated in countries that adopted DOTS, with 22 million lives saved.^2^

In view of the improvement in the epidemiological situation of TB attained through DOTS, WHO adopted SDGs (Sustainable Development Goals) in 2015. These aim to reduce the incidence by 80% and mortality by 90%, by the year 2030. Furthermore, WHO adopted the End TB Strategy with the objective of reducing TB incidence by 90%, by 2035, in comparison with 2015 estimates.^3^

The numbers continue to astound: WHO estimates that one third of the world’s population is infected with the tubercle bacillus. In 2010, 9.4 million new cases of TB were estimated, in addition to the existing 14 million cases. In that same year, there were 1.3 million deaths resulting from TB among people without AIDS worldwide. Another 380,000 deaths from TB occurred among HIV-positive patients.^4^

In Brazil, the numbers are slowly improving. In 1990, the national average was found to be 51.8 new TB cases per 100,000 inhabitants. This rate dropped to 38.4 in 2011.^5^ TB mortality also decreased over this period, from 3.6 to 2.4 deaths per 100,000 inhabitants.^5^ Because lack of reporting negatively affects TB statistics in this country, these numbers may be underestimated.^6^^,^^7^

Early diagnosing of suspected cases and proper medication-based treatment for carriers of pulmonary tuberculosis are the most important measures for controlling the problem.^8^ These measures are possible only with an active search for people who present the typical respiratory symptoms.^9^ This type of control is no easy task, since presence of a cough for more than two weeks is not a symptom that is specific to TB; it is also absent in 5% of pulmonary TB cases among adults.^10^

Public and private preventive medical services have failed to define measures that allow early diagnosing of the disease.^11^ Many studies have been conducted in different Brazilian cities and regions. The objective has often been to find the epidemiological patterns of TB in both the general population and in specific groups, such as children,^12^ adolescents,^13^ indigenous populations^14^ and the prison population.^15^

According to WHO, Brazil is among the 30 countries of the world with the worst situation of TB cases and TB/HIV cases.^3^ There were 81,137 notified TB cases in 2015, with approximately 5,500 deaths. The incidence was 41 per 100,000 inhabitants, and this is thought to correspond to 87% of the total number of cases, considering that the underreporting rate is estimated to be 13%. In relationship to multidrug-resistant TB, Brazil is in a comfortable situation compared with India and China, with incidence of 1.5% among all cases and 8% among retreatment cases.^3^

Knowledge of the epidemiological profile of TB is fundamental to the active search for cases and patients. It enables reduction of the times between the first symptoms, diagnosis and the start of supervised medication-based treatment.^16^^,^^17^ Higher mortality rates are associated with late diagnoses, which in turn are the result of failures in the organization of primary healthcare systems.^18^

Tuberculosis is a problem associated with poverty, and most studies have been conducted in lower-income cities. The present study took a different approach: a municipality with a high human development index (HDI) was chosen. The HDI is an indicator comprising three aspects of human development: life expectancy, education and per capita income. This indicator has been adapted in Brazil from the global HDI due to unavailability of data in this country; it is referred to locally as the Municipal HDI.^19^

## OBJECTIVE

The objective of this study was to characterize the TB cases reported in a municipality in the southeastern region of Brazil, from 2002 to 2011, based on demographic and clinical variables, as well as on the type of healthcare institution where the diagnosis was made.

## METHODS

This was an epidemiological study on incidence that was exploratory and analytical in nature. It used different time series and included all new cases of TB among residents of the municipality of Araraquara, São Paulo, that were reported to the Brazilian Notifiable Diseases Information System (SINAN) from 2002 to 2011. The study relied on this number of new TB cases reported during this period.

The municipality of Araraquara is located in the central region of the state of São Paulo. In 2010, it was found to have the 14^th^ highest HDI in Brazil and the 8^th^ highest in the state of São Paulo. Other municipalities in the region have similar characteristics. They include Ribeirão Preto, São Carlos and Rio Claro, all of which have more than 200,000 residents and relatively high HDIs. For this reason, this study of tuberculosis in Araraquara was expected to provide knowledge that might also aid other cities in the region.

The data used was obtained from the digital archives of the Special Healthcare Center of Araraquara (SESA), which is part of the University of São Paulo (USP). The name of the software is the Juarez System: the Integrated Public Health Information and Management System (referred to locally as Sistema Juarez). This database includes reports of TB cases among Araraquara residents. It has the same information as sent to SINAN and, in addition, it has information about the healthcare institution where the diagnosis was made. Population data was obtained from the Brazilian Institute for Geography and Statistics (IBGE) and was used to calculate the incidence rate of tuberculosis over the study period.^20^

The number of TB cases reported and population data from each year between 2002 and 2011 were collected. This period was analyzed firstly as a single unit and was then divided into two five-year periods (2002-2006 and 2007-2011) in order to identify epidemiological trends. The variables analyzed were sex, age, education level, occupation, TB location, healthcare institution where the diagnosis was made and presence of any other infectious diseases.

Statistical analysis was performed, and this included calculation of the incidence rates according to sex and age for each period. The two independent rates were compared using the ratio between them as point values (R) and 95% confidence intervals (95% CI).^21^

The results were displayed in tables. The criterion for deciding whether a significant difference existed between the rates in the five-year periods was to ascertain whether the confidence interval included the value 1. Thus, if this value was included, there was no significant difference. If the value 1 was not included, it could be said that the rates were significantly different.

## RESULTS

Between 2002 and 2011, 533 TB cases were reported among residents of the municipality of Araraquara, with an incidence of 26.82 cases per 100,000 inhabitants.

Through dividing the data into two periods of TB occurrence (2002-2006 and 2007-2011), decreased TB incidence was observed in the second period. It decreased from 30.24 to 23.60 cases per 100,000 inhabitants, as shown in Table 1. In the period 2007-2011, TB incidence was 22% lower than in the period 2002-2006. The first period exhibited significantly greater incidence of TB than the second period: R = 1.28 (95% CI: 1.06-1.50).

**Table 1.**
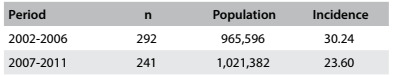
Number of reported cases of tuberculosis (n), population and incidence (per 100,000 inhabitants); Araraquara (SP), 2002-2006 and 2007-2011

According to sex, the majority of the cases were found to be among males (72.61%), as detailed in Table 2. Among males (Table 2), the ratio between the two periods was R = 1.30 (95% CI: 1.04-1.56), which was statistically significant. Different results were observed among females, for whom R = 1.22 (95% CI: 0.82-1.62), which was non-significant. In addition to the predominance of male patients, a statistically significant difference in incidence was found between males and females within each of the periods. In relation to the periods 2002-2006 and 2007-2011, respectively, R = 2.90 (95% CI: 2.80-3.00) and R = 2.73 (95% CI: 1.97-3.49).

**Table 2.**
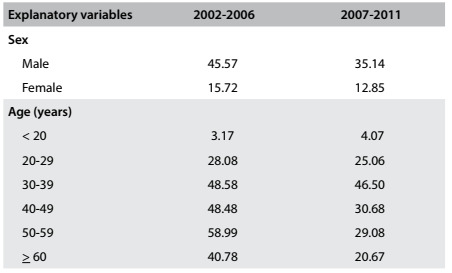
Incidence (per 100,000 inhabitants) of tuberculosis according to sex and age; Araraquara (SP), 2002-2006 and 2007-2011

Regarding age-specific incidence, it was found that in the period 2002-2006, the greatest incidence of TB was among the group aged 50-59 years. On the other hand, in the period 2007-2011, the greatest incidence was among the group aged 30-39 years. Comparison between these two periods showed an equilibrium in the specific incidence ratios up to 39 years of age. Starting at 40 years of age, a decrease in incidence was found in the period 2007-2011, relative to the period 2002-2006. The incidence ratios organized according to age between the two periods (2002-2006 and 2007-2011) were found to be as follows: R = 1.58 (95% CI: 0.98-2.18) for 40-49 years of age; R = 2.06 (95% CI: 1.16-2.90) for 50-59 years of age; and R = 1.97 (95% CI: 1.05-2.09) for 60 years of age and over. There was a significant decrease in incidence among residents aged 50 years and over.

Correlation of TB cases with educational level showed predominance of patients with only elementary school education, as can be seen in Table 3. These patients with elementary school education represented 62.33% of the cases in the period 2002-2006 and 57.68% of the cases in the period 2007-2011. These were followed by patients with complete or partial high school education, who represented 13.36% of the cases from 2002 to 2006 and 20.33% of the cases from 2007 to 2011. The third highest association was with illiterate patients, who represented 7.19% of the cases from 2002 to 2006 and 3.73% of the cases from 2007 to 2011, and these were followed by patients with tertiary education, who represented 5.48% of the cases from 2002 to 2006 and 4.56% of the cases from 2007 to 2011. The proportions with lack of information regarding educational level were 11.64% and 13.69%, respectively regarding the start and end of the subjects’ education.

**Table 3.**
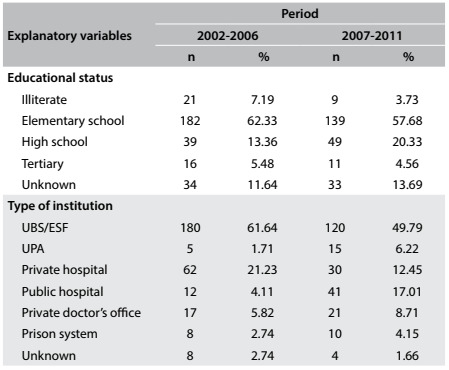
Patients’ educational status and type of institution where diagnosis was made, as number (n) and percentage (%); Araraquara (SP), 2002-2006 and 2007-2011

UBS = primary care units (Unidade Básicas de Saúde, UBS); ESF = family healthcare centers (Estratégia Saúde da Família, ESF); UPA = urgent care centers (Unidade de Pronto-Atendimento, UPA).

Considering the type of institution where the tuberculosis diagnosis was made, most diagnoses between 2002 and 2011 were made within Brazil’s public primary care network (Table 3). This network included primary care units (Unidades Básicas de Saúde, UBSs), family healthcare centers (Estratégia Saúde da Família, ESFs) and the School Healthcare Center of the state health department at the University of São Paulo (SESA). In the first period, 61.64% of the diagnoses were made within the primary care network, and this rate decreased to 49.97% in the second period. From 2002 to 2006, 21.23% of the diagnoses were made in urgent care centers or emergency rooms within private hospitals, and this proportion decreased to 12.45% in the second period. Also, 5.82% of the diagnoses were made in private doctors’ offices and this rose to 8.71% in the period from 2007 to 2011. In the second period, the diagnoses made within the public primary care network represented nearly half of the total number of diagnoses (49.79%), followed by diagnoses made in public hospitals (17.01%), urgent care centers or emergency rooms within private hospitals (12.45%) and private doctors’ offices (8.71%). The diagnoses made in public urgent care centers (UPAs), which are part of the Brazilian public healthcare system (Sistema Único de Saúde, SUS), represented only 1.71% of the diagnoses in the first period, and increased to 6.22% in the second period (Table 3).

The diagnostic methods used were clinical examination, bacilloscopy, sputum culture and X-ray, in all the healthcare institutions considered (UBSs, ESFs, SESA and public and private hospitals).

Regarding the type of conclusion from the treatment, cure rates of more than 70% were found in both periods. In the period 2002-2006, treatment conclusion due to death was more frequent (13.01%) than in the period 2007-2011 (9.13%). Meanwhile, treatment conclusions resulting from patient withdrawal showed the opposite trend, with a higher rate among patients in the second period (4.15%) than in the first (2.74%).

**Figure 1.**
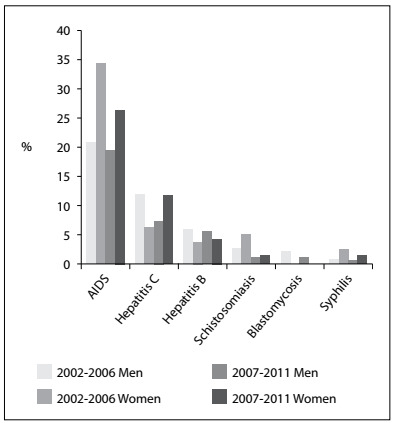
Comorbidities among tuberculosis patients in relation to sex, as percentage (%); Araraquara (SP), 2002-2006 and 2007-2011.

The most common coinfections among TB patients were found to be AIDS and hepatitis C, as shown in Figure 1. The incidence of AIDS decreased by 23% among women, dropping from 34.62% in the period 2002-2006 to 26.47% in the period 2007-2011. On the other hand, among men, the co-prevalence of AIDS remained stable, with rates of 21.03% in the first period and 19.65% in the second period. Concomitant incidence of TB and hepatitis C increased by 83.46% among females: the rate jumped from 6.41% in the first period to 11.76% in the second period. Among males, concomitant occurrence of TB and hepatitis C decreased by 38.19%: from 12.15% in the period 2002-2006 to 7.51% in the period 2007-2011.

Regarding the location of TB, 94.8% of the cases were pulmonary, while the remaining cases were extrapulmonary, involving the pleura, lymph nodes and larynx, along with cases of tuberculous meningitis and TB in other locations. Only two reports were cases of multidrug-resistant tuberculosis.

In relation to the occupations of TB patients, “homemaker” and “inactive” (people whose incomes are inconsistent and often zero) were the most common, representing 12.57% and 18.57% of the cases, respectively. Professions requiring low levels of specialization, such as handyman, construction worker and construction worker’s assistant, were also found, although less frequently. From 2002 to 2006, 2.74% of the patients were students and 2.40% reported having professions requiring higher education, such as doctors, lawyers and engineers.

## DISCUSSION

In a systematic review of the literature, TB was found to be associated with alcoholism, coinfection with HIV, low education level, marital status and low income.^22^ The municipality of Araraquara is located in a region with high HDI and, for this reason, exhibits a unique epidemiological profile for TB. In some ways, however, its epidemiological profile is similar to that of other, less economically and socially developed regions of Brazil.

From 2002 to 2011, the TB incidence was 26.82 per 100,000 inhabitants. It decreased over the course of this period: from 30.24 per 100,000 inhabitants (2002-2006) to 23.60 per 100,000 inhabitants (2007-2011). These rates are lower than the overall incidence of TB in Brazil, which was 36.7 per 100,000 inhabitants from 2008 to 2011.^23^^,^^24^ The decrease in the incidence of TB in Araraquara between the two five-year periods (2002-2006 and 2007-2011) was statistically significant. This finding demonstrates the trend towards real improvement in the status of this disease, which has resulted from changes to local social and sanitation conditions.

The predominance of males among TB patients (72.61% from 2002 to 2011) was consistent with the rates observed in other locations, such as in the entire state of São Paulo^25^ and the state of Minas Gerais, also in Brazil.^26^ In Araraquara, this pattern was found to be stable, with no statistically significant changes over the course of the period studied. On the other hand, it was higher than what has been observed around the world (62%). However, it is possible that the estimated incidence in other countries could also have been underreported.^3^

The age range with the highest incidence of TB cases from 2002 to 2006 was 50 to 59 years of age. The average age among the cases was lower in the period 2007-2011, such that the greatest incidence was among patients between 30 and 39 years of age. In the second period (2007-2011), there was also a significant decrease in cases among residents 50 years of age and over. This predominance of new cases among adults in younger age groups was similar to the findings reported in studies at other locations and over the same period. In the city of Ribeirão Preto, which is located in the same region and has a socioeconomic profile similar to that of Araraquara, the maximum incidence in 2006 occurred among young adults (from 20 to 39 years of age).^27^ Meanwhile, in the state of Minas Gerais, the residents most commonly affected between 2002 and 2009 were those between 20 and 49 years of age.^26^

Worldwide, 10% of TB cases were found among children under 14 years of age, which was concordant with Brazilian patterns in 2015.^3^ In Araraquara, in the first period (2002-2006), 3% of the cases occurred among people under 19 years of age, and this rose to 4% in the second period (2007-2011). These values indicate the good social and health structure of the municipality studied. It is difficult to analyze this difference and it could be considered to be a limitation of this study.

In the two periods considered in this study, predominance of patients with low educational levels was found, such that two thirds of the patients reported only having elementary school education. This finding is similar to the reports found in a review of the literature on this topic.^22^

The type of conclusion from the treatment was considered, and cure rates greater than 70% were found in both periods. This was similar to the national average reported in 2013.^28^ This consistency suggests that the higher HDI of the location studied has not been accompanied by greater efficacy among the measures taken to control TB.

Comparison of the locations of the diagnoses in the two periods showed some changes between the first and second periods. In the first period, 61.64% of the diagnoses were made within the primary care network (UBSs and ESFs), and this rate decreased to 49.97% in the second period. In the first period, the second most common location for the diagnosis was in private hospitals (21.23%). On the other hand, in the period 2007-2011, the second most common location for the diagnosis was a state public hospital that was established at the beginning of the twenty-first century in a neighboring city; this hospital accounted for 17.01% of the diagnoses, and private hospital diagnoses decreased to 12.45%. This partial replacement of the primary care network by a public hospital is likely to be unique to this city. The public hospital in question is state-run; it is managed by the University of São Paulo. Because of the high quality of its services and its perceived prestige among local residents, this hospital became the preferred option for healthcare among the population. This hospital has also become a common destination for patients transferred by physicians within the public primary care network.

From 2002 to 2011, the most common locations for TB diagnoses within the city of São Paulo were hospitals and emergency rooms.^29^ In the city of Ribeirão Preto, 57.4% of TB diagnoses were made within urgent care services in 2009;^16^ similarly, in the city of São José do Rio Preto, 49.4% of the diagnoses in the same year were made within urgent care services.^30^

Coinfection with AIDS was found to have decreased by 23% among females when the two periods were compared: from 34.63% (2002-2006) to 26.47% (2007-2011). This coinfection with AIDS remained stable and lower among men, with rates of 21.03% in the first period and 19.65% in the second period. In Ribeirão Preto, the average rate of coinfection with AIDS for both sexes was 25% in 2006.^27^ The numbers found in Araraquara and its region are very different from the national average, which was 10.1% (both sexes) in 2013; national rates among men are higher in general.^28^ Although WHO recommends serological evaluation for HIV among TB patients, it is not done routinely in Araraquara, and only 55% of these patients worldwide underwent this evaluation in 2015.^3^

Only limited data on other comorbidities associated with TB are available in the literature, which makes additional comparisons difficult. In the present study, an 83.46% increase in TB-hepatitis C comorbidity was found among females, which reached a rate of 11.76% in the period 2007-2011. Among males, comparison between the two periods showed a 38.19% decrease in this comorbidity: from 12.15% to 7.51%. To understand these hepatitis C trends (the increase among women and the decrease among men), further study is required.

Occupations requiring less specialization (and, therefore, resulting in lower incomes) were found to be most common among the TB patients, who most commonly reported being homemakers or inactive (people whose income is often zero); or being construction workers or construction workers’ assistants. This pattern is similar to what has been reported in the literature, such as in the municipality of Ribeirão Preto, where TB was found to be associated with low income.^31^

It should be mentioned that this study may present some limitations due to the high drop-out rate from TB treatment, and to the lack of precise information on education. These occurrences may have compromised the findings regarding the epidemiological and clinical profile, with a greater lack of information in the second period.

It could be observed that, over the 10-year period, no population movements occurred in relation to immigration or wars, and there were also no changes in public health policies and healthcare systems that could have affected the rates of new TB cases, and consequently, the TB control programs. The literature shows that, in Brazil, tuberculosis is not a public health problem restricted to areas with less human or social development. The present study, conducted in a municipality with high HDI, demonstrates this fact. The similarities and differences between the municipality of Araraquara and the national trends in Brazil, in terms of epidemiological and clinical patterns, should serve as a basis for creation of public policies and disease control measures, even in municipalities with higher HDIs. The more general objectives are always faster diagnosis for the disease, increased cure rates and greater priority towards making resources available for social groups within which the disease is most frequent.

## CONCLUSIONS

The incidence of tuberculosis in this municipality was lower than the national incidence, with a declining trend and a high cure rate, and the main coinfections were AIDS and hepatitis C.
